# Effects of overnight military training and acute battle stress on the cognitive performance of soldiers in simulated urban combat

**DOI:** 10.3389/fpsyg.2022.925157

**Published:** 2022-07-26

**Authors:** Tomi Passi, Kristian Lukander, Jari Laarni, Johanna Närväinen, Joona Rissanen, Jani P. Vaara, Kai Pihlainen, Kari Kallinen, Tommi Ojanen, Saija Mauno, Satu Pakarinen

**Affiliations:** ^1^Finnish Institute of Occupational Health, Helsinki, Finland; ^2^VTT Technical Research Centre of Finland Ltd., Espoo, Finland; ^3^Savox Communications Oy Ab, Espoo, Finland; ^4^Department of Leadership and Military Pedagogy, National Defence University, Helsinki, Finland; ^5^Training Division, Defence Command, Helsinki, Finland; ^6^Finnish Defence Research Agency, Finnish Defence Forces, Tuusula, Finland; ^7^Department of Psychology, Faculty of Social Sciences (Psychology), and University of Jyväskylä, Tampere University, Jyväskylä, Finland

**Keywords:** military, stress, cardiac autonomic activity, response inhibition, cognitive performance, vigilance, sustained attention, sleep loss

## Abstract

Understanding the effect of stress, fatigue, and sleep deprivation on the ability to maintain an alert and attentive state in an ecologically valid setting is of importance as lapsing attention can, in many safety-critical professions, have devastating consequences. Here we studied the effect of close-quarters battle (CQ battle) exercise combined with overnight military training with sleep deprivation on cognitive performance, namely sustained attention and response inhibition. In addition, the effect of the CQ battle and overnight training on cardiac activity [heart rate and root mean square of the successive differences (RMSSD)] during the cognitive testing and the relationship between cardiac activity and cognitive performance were examined. Cognitive performance was measured with the psychomotor vigilance task (PVT) and the sustained attention to response task (SART). Altogether 45 conscripts participated in the study. The conscripts were divided into control (CON) and experimental (EXP) groups. The CON completed the training day after a night of sleep and the EXP after the overnight military training with no sleep. Results showed that the effect of the overnight training on cognitive performance and the between-group difference in heart rate (HR) and heart rate variability (HRV) depended on the cognitive test. Surprisingly, the cognitive performance was not largely affected by the CQ battle. However, as expected, the CQ battle resulted in a significant decrease in RMSSD and an increase in HR measured during the cognitive testing. Similarly, the HR parameters were related to cognitive performance, but the relationship was found only with the PVT. In conclusion, fatigue due to the overnight training impaired the ability to maintain sufficient alertness level. However, this impairment in arousal upregulation was counteracted by the arousing nature of the SART. Hence, the conscripts' cognitive performance was mainly preserved when performing a stimulating task, despite the fatigue from the sleep loss of the preceding night and physical activity.

## Introduction

In safety-critical occupations, such as rescue and recovery, police, and military, maintaining a high level of performance in challenging or even adverse conditions is critical (Krueger, 1989). As the operative duties can vary from long-term passive surveillance or monitoring to active or even high-tempo operations, one needs to sustain and adapt one's focus of attention and decision-making according to the situation and task at hand. Moreover, in demanding situations, these cognitive capabilities can be compromised by either the acute stress evoked by the operative task and/or prolonged stress and strain from continuous physical activity, mental load, energy deficit, and sleep loss (Lieberman et al., 2005; Suárez and Pérez, 2013; Taverniers and De Boeck, 2014; Giessing et al., 2019). This study examined the effects of military overnight training with sleep deprivation and acute stress from close-quarters battle (CQ battle) exercise on the cognitive performance of a soldier. Cognitive performance was assessed using two tasks: a monitoring task resembling, for instance, radar monitoring and an active decision-making task that could be compared to, for example, a decision to shoot or not to shoot.

The function of the stress reaction is to energize and increase the psychological and physiological readiness to respond. Stress reaction emerges when demands tax or surpass one's resources or endanger an individual's wellbeing (Lazarus and Folkman, 1984). During the stress reaction, the balance in the autonomic nervous system (ANS), which consists of the sympathetic nervous system (SNS) and parasympathetic nervous system (PNS), is altered resulting in multiple hormonal changes and increased arousal. At the fight-or-flight stage (i.e., acute stress stage), catecholamines are secreted in the circulation due to the increased activation of the sympathomedullary pathway (SAM), a part of the SNS (Gunnar and Quevedo, 2007). This results in dilation of pupils, acceleration of the heart and lung activity, and heightened sensitivity to threat-related cues (Chu et al., 2021). When the situation is perceived as safe, the autonomic homeostasis is restored *via* increased activation of the PNS, while the SNS activation is inhibited. The Yerkes-Dodson law (1908) states that a sufficient vigilance or energetic level is needed to retain an appropriate level of focus on any given task. However, excessive or minimal arousal often negatively affects the performance (Yerkes and Dodson, 1908; Arnsten, 2009).

Increased fatigue induced by sleep loss is closely related to a lower vigilance level (Killgore, 2010), which becomes evident even after one night without sleep (Haslam, 1984). According to the state instability hypothesis, sleep loss causes momentary attentional lapses due to variation in an alert state (Doran et al., 2001). In other words, individuals with sleep loss lack the energetic resources to upregulate their alertness to show stable performance. This effect can be described from a neuropsychological perspective. The frontal areas of the brain, critical not only for executive functioning but also for top-down control of the vigilant state, are the first to suffer from sleep loss (O'Connell et al., 2008). Perhaps the most consistent finding is that slowing in processing speed, manifested in prolonged reaction time (RT), is a hallmark of low vigilance caused by sleep loss (Doran et al., 2001).

Simulated combat scenarios have been successfully employed to induce high acute operational stress (Lieberman et al., 2005; Suárez and Pérez, 2013; Taverniers and De Boeck, 2014; Giessing et al., 2019). An increase in stress, anxiety, and mental effort, as well as impairment in shooting accuracy, have been found in law-enforcement reality-based training utilizing both low-stress (mannequin) and high-stress (human) targets (Murray, 2004; Taverniers and De Boeck, 2014; Giessing et al., 2019). Military training in harsh environmental conditions that included sleep loss has been shown to result in decreased vigilance, increased fatigue, lowered mood, and decreased higher cognitive abilities (Lieberman et al., 2005). According to Hockey (1997), fatigue, in low controllability and high environmental demand situations, is related to SAM activation due to direct engagement with an acute stressor (i.e., active problem-focused coping). Suárez and Pérez (2013) found increased SNS activation in urban combat training even when physical activity was at a low level. According to the authors, the psychological stress caused by the uncertainty regarding the location of the threats led to fatigue impairing post-combat information processing ability.

Sustained attention to response task (SART) and psychometric vigilance task (PVT) are commonly used to study psychomotor processing, particularly, sustained attention, vigilance, response inhibition, and arousal (Robertson et al., 1997; Drummond et al., 2005; McIntire et al., 2014). In PVT, the participant is required to react as fast as possible to a rarely and infrequently presented stimulus. In this rather monotonous task, the performance is highly dependent on the vigilance level of the participant, and not so much on higher cognitive functions (McIntire et al., 2014). In contrast, in go/no-go tasks, such as SART, the aim is to respond to repetitive target stimuli (“GO”) but not to the rarely presented non-target stimuli (“NO-GO”). For this, one needs to overcome the motor response routine through inhibition. This exerts demands on higher-order frontal lobe functions, also known to suffer from the effect of stress and fatigue (Boksem and Tops, 2008; Arnsten, 2009; Lim and Dinges, 2010). Sleep loss has been shown to prolong response times and increase inhibition errors in go/no-go tasks (Chuah et al., 2006; Drummond et al., 2006; Shao et al., 2009; Rabat et al., 2019); also refer Skurvydas et al., 2020 and Hudson et al., 2020a for conflicting findings; Magnuson et al., 2022. Moreover, not only the impairing effects of fatigue but also stress can remain well after the presentation of the stressor. For example, Alomari et al. (2015) showed that acute stress impairs SART performance up to 30 min after stress induction. On the other hand, stress-induced arousal, and the related elevation of the sympathetic tone of the ANS may act as an antagonist for drowsiness- and boredom-related low activation improving the performance in PVT, particularly in individuals suffering from sleep loss (Smith and Nutt, 1996).

Psychophysiological assessments offer an objective method to quantify stress and mental effort (Fairclough and Mulder, 2012; Kim et al., 2018). Heart rate variability (HRV) is commonly used to index the alternated activity between the SNS and PNS. Low HRV has been linked to high SNS activation and/or PNS withdrawal (Shaffer et al., 2014). The root mean square of successive differences (RMSSD), a metric of beat-to-beat variability, is often applied to quantify short-term HRV (Munoz et al., 2015). RMSSD depicts the activity of the vagus nerve (Shaffer et al., 2014), that is, the activity of the PNS. Furthermore, RMSSD is relatively free from respiratory effects (Hill et al., 2009). In addition to the HRV indices, heart rate (HR) is connected to the alternating activity of the SNS and PNS so that SNS activation/PNS withdrawal is related to cardiac acceleration (Acharya et al., 2006).

The heart (functioning) is closely related to brain activity. According to the neurovisceral integration model (Thayer et al., 2009), the same neural networks involved in autonomic, emotional, and cognitive self-regulation also participate in cardiac autonomic activity control. Thayer et al. (2009) state that vagally mediated HRV is an index of an individual's ability to self-regulate and that higher HRV relates to greater adaptability and flexibility of responses. The relationship between high HRV and enhanced performance has been shown, especially in tasks requiring higher cognitive functions, such as working memory and attention (Hansen et al., 2003). Furthermore, high HRV has been associated with fast and accurate performance (Thompson et al., 2015). Hence, first, HRV recordings provide information on the regulation of the two branches of the ANS; second, better self-regulation, reflected in higher HRV, is associated with a better performance in tasks demanding complex cognitive abilities; and third, cardiac recordings can be utilized to examine the relationship between the ANS activity and performance in cognitive tests.

There is a limited understanding of the overlapping effects of stressors such as sleep loss and physical activity and acute stress on military performance. The operative stressors such as sleep loss and sustained physical activity typically decrease vigilance and performance. On the other hand, acute stress may either energize and increase vigilance or, when excessive, undermine cognition. Therefore, this study examined the effects of acute stress evoked by the CQ battle either with (EXP) or without (CON) sleep loss on arousal, vigilance, and decision-making in a field setting. The cognitive performance was measured using PVT and SART as they capture mental abilities relevant to many occasions in safety-critical fields. Situations can rapidly switch from passive surveillance to a fast tempo-operative activity. The former may require the detection of subtle environmental changes and arousal upregulation while the latter demands quick decision-making. The PVT can be considered a monitoring task, indicating arousal and attentional state (Drummond et al., 2005; McIntire et al., 2014), relevant, for instance, to a radar monitoring task. On the other hand, the SART is more of an active decision-making task, requiring higher cognitive abilities, such as response control and inhibition (Robertson et al., 1997), and comparable to, for instance, a shooting-decision task (go-no-go).

First, it was hypothesized (H1) that the performance of those participants who completed the overnight training (EXP) would be impaired due to decreased arousal and vigilance and reduced self-regulative capacity, compared with their well-rested counterparts (CON) during the first SART and PVT test (preTest, before the CQ battle). This would become evident during the SART as in prolonged RT and increased percentage of error of commissions (EoC) and during the PVT in prolonged RT and an increased number of attention lapses. Second, the CQ battle was hypothesized (H2) to result in high acute stress during the cognitive testing, impairing the performance of both groups in the SART with the mentioned performance measures (midTest and postTest vs. preTest, i.e., after vs. before the CQ battle). Again, EXP was expected to perform worse than CON during the SART tests after the CQ battle (midTest and postTest). On the other hand, during the PVT (postTest), EXP performance would be protected by battle-induced stress and arousal. Therefore, EXP performance would reach the level of CON measured with PVT RT, and attention lapses after the CQ battle. In addition, it was hypothesized (H3) that the CQ battle-related high acute stress would become evident in HR and RMSSD (i.e., decreased RMSSD and increased HR) during the SART (midTest and postTest vs. preTest) and PVT (postTest vs. preTest). Furthermore, EXP participants' RMSSD was expected to be lower and HR higher than that of CON participants during each SART and PVT tests. Lastly, higher PNS activity was hypothesized (H4) to be associated with enhanced executive functioning, e.g., increased flexibility in responding. On the other hand, low PNS activity was expected to reflect greater alertness and wakefulness (hyperarousal). Therefore, it was hypothesized that higher RMSSD and lower HR would be related to better performance, as indicated by shorter mean RT and decreased EoC during the SART. During the PVT, increased HR and lowered RMSSD was expected to be associated with a shorter median RT and fewer attention lapses.

## Materials and methods

### Participants

Fifty-five conscripts [49 males and 6 females; (*Mage* = 20.10, *SD* = 0.96)] from the Finnish Defense Forces gave their informed consent and took part in baseline measurements. Ten participants withdrew from the study (due to illness or suspected illness) before the military training phase. Thus, the final data set consisted of 45 participants who all completed the entire study. The age of these participants varied from 19 to 24 years (*Mage* = 20.14, *SD* = 1.04).

The participants signed the written informed consent. The study was approved by the Ethics Committee of the Hospital District of Helsinki and Uusimaa and the Finnish Defense Forces (AQ9520). The study protocol followed the Declaration of Helsinki.

### Procedures

**Figure 1:**
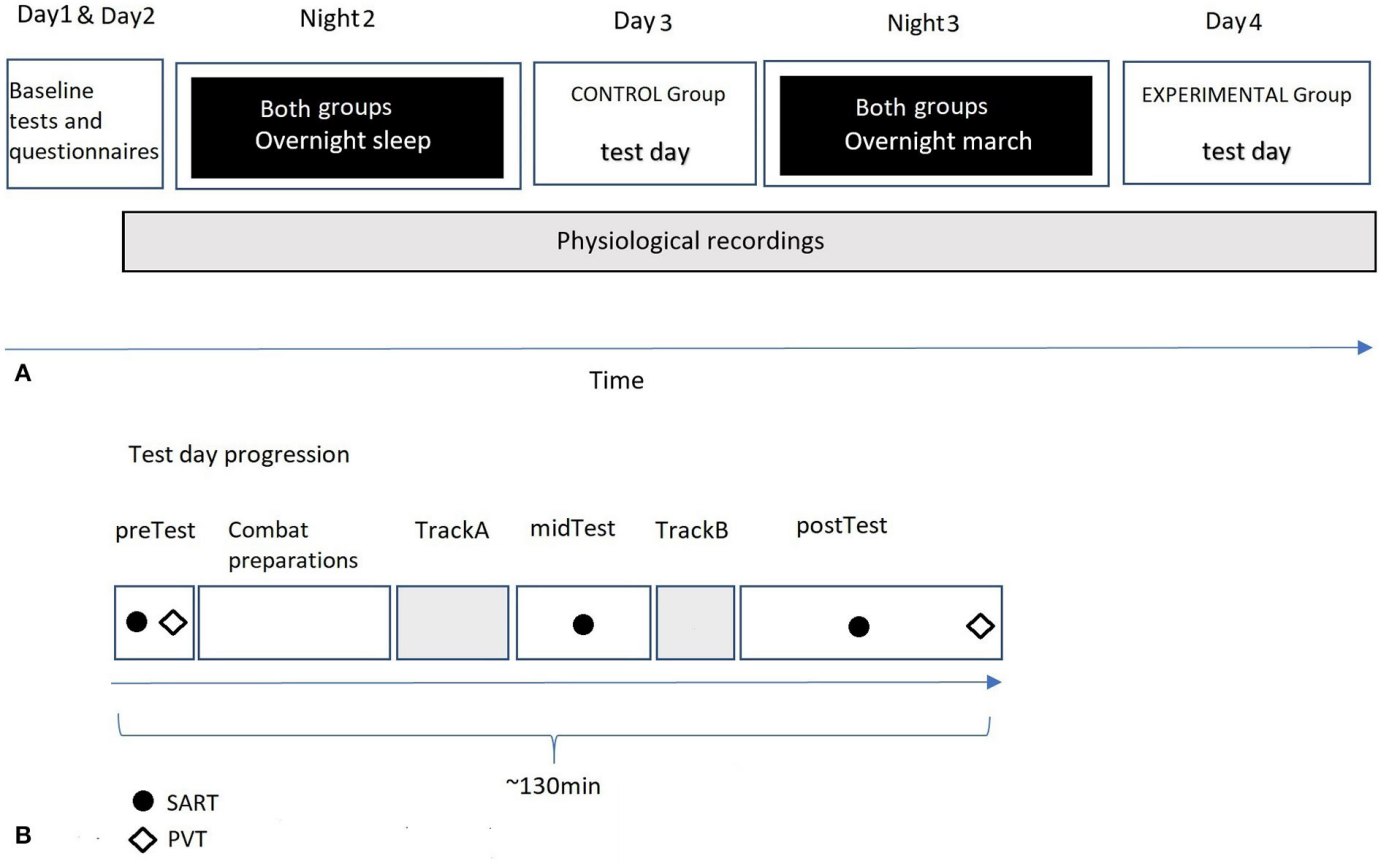
**(A)** The temporal order of the main events throughout the whole study. **(B)** A three-person drill team's test day procedure. The sustained attention to response task (SART) and psychometric vigilance task (PVT) were performed in the order displayed in the picture with a comparative in-between task duration.

The study included three consecutive parts: baseline, normal sleep (CON) or overnight military training with sleep loss (EXP), and close-quarters battle exercise (CQ battle; Figure 1A).

### Baseline measures

Before the actual military field training, baseline information was collected. To ensure that the study groups are comparable with respect to the key characteristics that may affect the study results (such as cognitive ability, fitness, age, and cardiac function) or in case differences were found, that can be taken into account in the modeling approach. The baseline measurements collected during the daytime included questionnaires, surveys, and cognitive testing, while the baseline measures for HR were collected during the first night.

On the first day of the study, 37 participants filled out surveys and questionnaires and performed several tablet tests, including the SART, as baseline measures in a classroom setting. Due to practicality issues, the remaining 18 participants performed these on the day after. The background questionnaire included basic demographic information (e.g., age, gender, handedness, and education), information on body mass index (BMI), health, and possible medications (for safety and interpretation of physiological data), as well as their self-reported scores in a shooting test and 12-min running test (to evaluate possible differences in the shooting performance and cardiorespiratory fitness).

After the baseline tests on day 2, the participants were equipped with Movesense^TM^ Heart Rate sensors (Suunto Oy, Vantaa, Finland) attached to a chest strap. The sensors were connected to a portable router, which recorded the HR data *via* Bluetooth. Baseline HR was collected during the night following the first study day. The overnight HR measures were filtered to include data only when the participant was in a supine position. This was based on Link GT9X (ActiGraph LLC, Pensacola, FL, USA) posture data; thus the duration of the measure varied from individual to individual (range: 4.35–8.5 h). In cases with missing Link GT9X-data, the duration of the overnight HR was collected from self-reported hours in bed.

### The course of the experiment

The final set of participants, 45 conscripts, were pseudorandomized into two groups (i.e., EXP and CON) by a drill instructor. The participants were further divided into three-person drill teams. CON (7 teams) performed the CQ battle the day after all the baseline measures were conducted. Both groups (15 teams) began an overnight training the same evening when CON had completed the CQ battle. The distance marched during the overnight training from 10:00 p.m. to 06:00 a.m. was approximately 30 km. EXP (8 teams) performed the CQ battle after completing the overnight march (Figure 1A). Using this arrangement, all participants had similar training. The only difference was that CON had an uninterrupted overnight sleep before the CQ battle while EXP completed the CQ battle sleep-deprived after the overnight military training. Moreover, as the study was conducted as a part of the urban combat military field training within the military service, the drill teams participated in various training activities in addition to the CQ battle. Thus, all the drill teams had mentally and physically similar activities during the test day when not in the CQ battle. The provided activities also ensured that the participants stayed active, preventing any opportunity for sleep before the CQ battle.

### Close-quarters battle exercise

**Figure 2:**
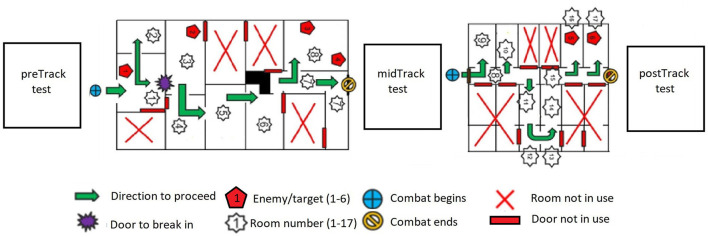
Overview of the main activities and the urban buildings included in the experiment.

The main tasks of the CQ battle were to break into a building, notice any wounded and give first aid, clear an urban building from the enemies, and command and capture individuals who did not show resistance. The CQ battle consisted of two simulated urban buildings (Figure 2). The first track (Track A; the duration ~15 min) was considered more demanding than the second track (Track B; the duration ~10 min). Both the soldiers and the enemies were armed with Glock FX^TM^ training pistols with short-range color-marking training ammunition. The use of these weapons increased the realism of the CQ battle as the projectiles likely caused some sensations of pain when hitting the target. Although the conducted simulation was not physically demanding *per se*, the CQ battle setting induced high acute stress for the participants. The soldiers progressed from one room to another at a slow or very slow walking pace. Therefore, changes in heart rate during the training scenarios were considered to arise predominantly from the stressfulness of the simulated situation.

The CON started the battle day at approximately 8:30 a.m., and the EXP started at 7:30 a.m. The drill teams entered the first testing (preTest) in groups of two- or three-person drill teams and started the testing and the CQ battle stepwise. Each drill team performed the first battery of cognitive tests and surveys, after which they made the required preparations before entering the building (e.g., preparing the equipment and receiving instructions from a drill team leader) (duration ~40 min). After mounting the equipment, the drill team entered Track A. When the CQ battle, including the three testing sessions, was performed, the drill team unmounted unnecessary equipment and left the training area as a team (Figure 1B). Simultaneously, when another drill team entered Track A, the following drill team proceeded to the preTest. The drill team from the preTest was allowed to enter Track A after the drill team ahead had left the midTest, and Track A was ensured to be empty. This sequence went through until all the drill teams had performed each section included in the test day.

The SART was performed before, in the middle of, and after the CQ battle, and the PVT before and after the CQ battle. During the testing, the participants sat supervised around a table, and instructions for each test and questionnaires were given. The participants in each drill team were instructed to begin each test simultaneously.

The SART and PVT, and two questionnaires (Karolinska Sleepiness Scale [KSS; Shahid et al., 2011] and NASA Task Load Index questionnaire concerning SART [NASA-TLX; Hart and Staveland, 1988]); duration ~ 3 min), were completed using a tablet during the preTest (duration ~ 20 min). The midTest (duration ~ 15 min) consisted of the SART (starting ~10 min after Track A) and four questionnaires (Two NASA Task Load Index questionnaires, one concerning Trac kA and one SART [NASA-TLX; Hart and Staveland, 1988], Karolinska Sleepiness Scale [KSS; Shahid et al., 2011], and self-assessment Manikin (SAM) test (Bradley and Lang, 1994); duration ~ 5 min). After the midTest, the drill teams were prepared for Track B. In the postTest (duration ~ 45 min), the SART began after approximately 15 min of the testing, and the PVT was performed as the last task during the postTest. In addition to the SART and PVT, postTest consisted of one other test (Visual Search Task [Motter and Simoni, 2008]; duration ~ 5 min) and nine questionnaires (A questionnaire considering details of the track, Two NASA task load index questionnaires, one concerning Track B and one SART [NASA-TLX; Hart and Staveland, 1988], Karolinska Sleepiness Scale [KSS; Shahid et al., 2011], self-assessment Manikin [SAM] test (Bradley and Lang, 1994), perceptual and memory distortions questionnaire [created on the basis of Grossman, 2008], mission awareness rating scale [MARS; (Matthews and Beal, 2002)], team workload assessment scale [TWAS; (Lin et al., 2011)], and self-evaluation of participant's own performance (constructed in close collaboration with a military expert); duration ~ 20 min). The results of those tests and questionnaires other than SART and PVT are not reported here.

The SART and visual search task as well as all questionnaires were implemented in the Inquisit software package (Millisecond Software, Seattle, WA, USA) and performed using a tablet computer with a USB keyboard attached for responses. In SART, one is to respond to frequently presented digits from 1 to 9 appearing in a random sequence (go response) and refrain from reacting to infrequently presented targets (digit 3; no-go response). The importance of speed and accuracy was stressed. Participants were presented with an on-screen stimulus for 250 ms. After the stimulus was presented, there was a circle with an X on the screen for 900 ms during which the response had to be given. Hence, the interstimulus interval was 1,150 ms. The task was to respond by pressing a keyboard spacebar when a digit from 1 to 9 (excluding three) was presented (go) and refrain from responding when digit 3 was presented (no-go). The inability to refrain from pressing when digit 3 is presented is termed as an EoC. Each testing session included 200 go and 25 no-go stimuli. The EoC (%) and mean RT for the correct go-response (ms) were calculated for each participant from the SART data.

The PVT was performed using a simple handheld apparatus that included the Movesense button and LED light (Suunto Oy MoveSense, Vantaa, Finland). Participants were instructed to press the button as quickly as possible each time the LED light lit up. Interstimulus intervals varied randomly from 1 to 10 s. The duration of the task was 10 min. Median RT was used to index the level of vigilance and RTs exceeding 500 ms indexed attention lapses indicating attentional disengagement. The PVT responses were recorded on the Actilink wrist devices *via* Bluetooth connection.

### Heart rate measurement

Heart rate was measured throughout the whole experiment with the Suunto Movesense sensor (Suunto MoveSense Oy. Vantaa, Finland). The data was streamed to a combatant's portable Savox BS router via Bluetooth connection. The three-person drill team leader was carrying the router inside a combat vest throughout the study. In addition to the HR, the sensor registered a movement, skin temperature, and body position.

## Statistical analysis

### Group comparisons in baseline

All statistical tests were conducted using R version 4.0.5. (R Core Team, 2021). The between-group comparisons were conducted regarding age, gender, skill level, education, a 12-min running test, the SART (EoC and mean RT), the PVT (median RT and attentional lapses), and heart rate parameters (RMSSD and HR) to determine whether the groups were comparable in these terms. Due to the non-normality of the data, age, the SART, PVT, and the 12-min running test were compared using the unpaired two-sample Wilcoxon Signed Rank test for independent samples. The Chi-square test was applied to compare the groups regarding gender, skill level, and education. The SART and PVT, in the baseline, were tested on the day before the first battle exercise day. Similarly, the HR parameters for the between-group comparison were taken from the first night, over which both groups had a regular sleep. The *p*-values of the tests were Bonferroni corrected according to the number of tests conducted.

### The battle exercise day

The unpaired two-sample Wilcoxon Signed Rank test for independent samples was applied to study the differences between the groups in the preTest (SART, PVT, HR) before the CQ battle for all measures.

Second, we investigated the effect of the CQ battle on SART and PVT performance, and HR parameters at the midTest and postTest. Furthermore, we inspected if the groups differed in these parameters overall and in each testing session. The effect was studied with linear mixed models using lmerTest package (Kunzetsova et al., 2017). Three models were built separately for each response variable. In the first model, the testing session was added as a fixed effect and the participant as a random effect (random intercept model). The second model was built to study if there were between-group differences in these parameters. It included the group and testing session as fixed effects and the participant as a random effect (random intercept model). The third model was built to inspect if the group and the testing session interaction was present to find out if the groups differed during the testing sessions in the parameters of interest. In this model, the testing session, group, and their interaction were added as fixed effects and the participant as a random effect (random intercept model). Satterthwaite approximation (ANOVA function of the stats package) was applied to compare the three models. If the second model did not improve the fit significantly, the interaction was not investigated further. If the interaction improved the fit significantly in comparison to the second model, it was left as the final model. *Post-hoc* pairwise comparisons on the marginal means were conducted using the Tukey method implemented in the emmeans package in R (Lenth, 2021). When a significant main effect only of the testing session or both the main effects of the group and testing were present, the differences between the testing sessions were studied *post hoc*. When the group and testing session interaction was a significant addition to the model with both main effects, the differences between the groups during the testing sessions and the difference between the testing sessions within groups were studied *post hoc*.

### The SART—reaction time and errors of commission

Since speed-accuracy tradeoff is a well-known phenomenon in the SART (Peebles and Bothell, 2004; Helton, 2009; Seli et al., 2013; Dang et al., 2018), this effect was further explored between the groups to understand our results better. The effects of RT and group on EoC were examined with linear mixed models using lmerTest package (Kunzetsova et al., 2017). Mean RT, group, and their interaction were set as fixed effects and the participant as a random effect (random intercept model).

### HR parameters and sustained attention

Lastly, the effect of the HR parameters on the SART and PVT was examined with linear mixed models using lmerTest package (Kunzetsova et al., 2017) utilizing the whole sample. Since group was a significant predictor of the HR and RMSSD, the effect of the group was controlled in the analyses. First, all the participants were split into high and low HR and high and low RMSSD groups based on the median HR/RMSSD value of the three testing sessions of both groups. During the SART, the median HR was 87 bpm and the RMSSD was 30 ms. During the PVT, the median HR was 80 bpm and the median RMSSD was 50 ms. In the model, the HR parameter group (low/high) and group (CON/EXP) were fixed effects, and the participant was a random effect (random intercept model). Second, through visual scrutiny, it seemed that EXP participants in the high HRV and low HR groups had the longest RTs after the CQ battle. For this reason, we further investigated *post-hoc* to test if the effect was due to EXP in the third testing session or solely due to EXP using linear mixed models. We built two separate models employing backward elimination, and the fit was analyzed using Satterthwaite approximation (ANOVA function of the stats package). In the first model, group, HR parameter group, and testing session and their three-way interaction were the fixed effects and participants, a random effect. In the second model, group and HR parameter group and their interaction, and in addition, testing session were fixed effects and participants, a random effect. In case significant interaction effects were found, pairwise comparisons on the marginal means were conducted using the Tukey method implemented in the emmeans package (Lenth, 2021).

## Results

### Group comparisons in baseline

The groups did not differ in age, gender, skill level, education, or self-reported 12-minrunning test result after the *p*-value adjustment. However, before adjusting the *p*-value, the difference between the groups in the 12-min running test was significant (< 0.01, r = 0.40; moderate effect size). The median distance run by the EXP was 2,725 m (IQR = 241), while the median in the CON group was 2,600 m (IQR = 400). Furthermore, the groups did not differ in PVT median RT, attention lapses, or in SART errors of commission, and mean RT. Similar findings were found considering the HR parameters (RMSSD and HR) measured over the first night. The groups were comparable with respect to the HR measures. Since there was an indication of possible group differences concerning the 12-minrunning test, we inspected whether it was associated with the HR parameters, SART, and PVT during the baseline. No association between the 12-minrunning test and the mentioned variables was found. Furthermore, we used the 12-min running test as a covariate in the linear mixed models to explore if it affected the results that were obtained. The result showed that the 12-min running test as a covariate did not affect the results significantly (i.e., if an independent variable was a significant predictor, the significance did not become nonsignificant and vice versa). Hence, the results are provided without the covariate.

### The sustained attention to response task

#### Errors of commission

**Figure 3:**
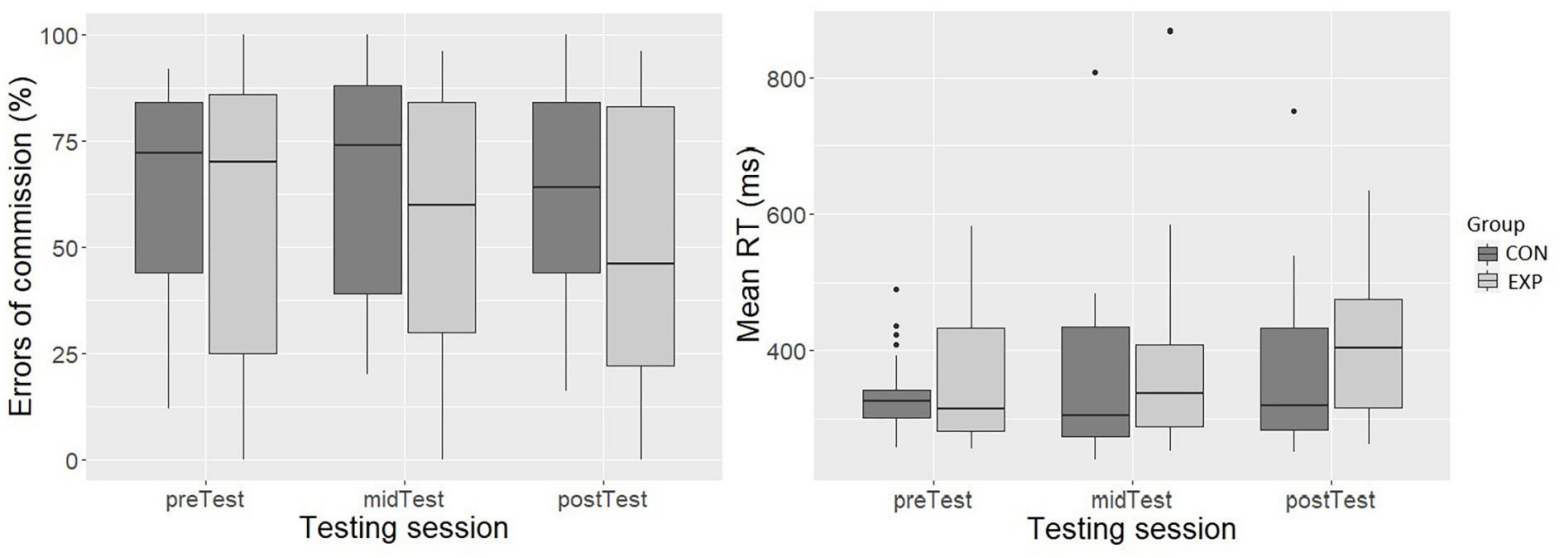
The error of commissions (%) (y-axis; left-side graph) and mean RTs (ms) (y-axis; right-side graph) during the SART testing sessions (x-axis; both graphs). The box depicts 50 % of all values (IQR). The top 25% and bottom 25% of all values are excluded from the box. The top and bottom of the whiskers are the minimum and maximum, excluding outliers (outliers defined 1.5 x IQR). The horizontal line inside the box represents the median value.

There were no differences between the groups during preTest EoC. Adding group did not improve the model fit; thus, the model with one predictor (testing session) was the final model. The testing session was not a significant predictor in the model. The EoC percentage of the groups was at a comparable level during each testing session, and in addition, no change in EoC was observed over the three testing sessions (Figure 3).

#### Mean RT

There were no differences between the groups during preTest in mean RT. Adding group did not improve the model fit; thus, the model with one predictor (testing session) was the final model. A significant effect of the testing session on mean RT was observed in the simpler model [*F*(2, 75.44) = 3.32, *p* < 0.05]. There was 38.03 ms (±14.91, 95% CI [8.81, 67.24]) slowing in mean RT during postTest compared with the preTest. The mean RT of the midTest did not differ from the preTest RT (Figure 3).

#### The SART mean RT and errors of commission

The mean RT had a highly significant effect on EoC [*F*(1, 111.55) = 50.03, *p* < 0.001]. The more there were EoC, the shorter was the mean RT (*b* = −0.09, ± 0.04, 95% *CI* [-0.14,−0.03]). In addition, a significant mean RT and group interaction was present [*F*(1, 111.55) = 5.34, *p* < 0.05]. The slope of EXP between mean RT and EoC was significantly steeper than that of CON. (*b* = −0.08, ± 0.04, 95% *CI* [−0.15, −0.01]).

### The psychomotor vigilance task

#### Median RT

**Figure 4:**
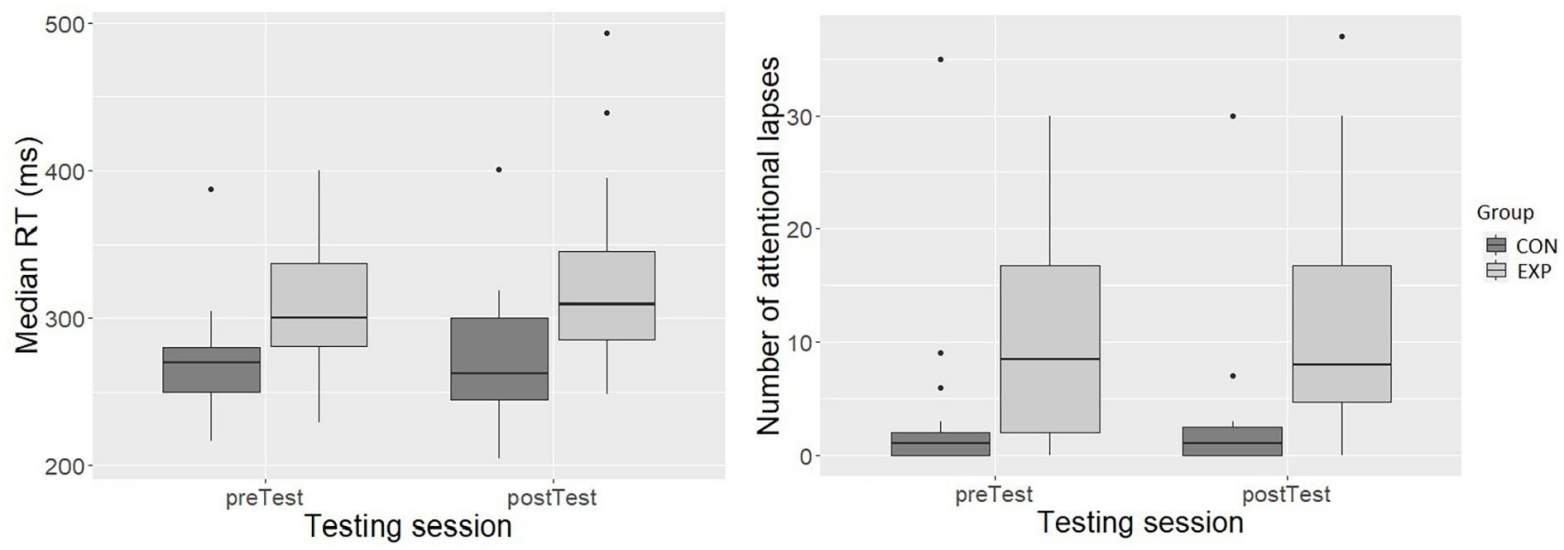
Median RT (ms) (y-axis; left-side graph) and number of attentional lapses (y-axis; right-side graph) during the two PVT testing sessions (x-axis; both graphs). The box depicts 50 % of all values (IQR). The top 25% and bottom 25% of all values are excluded from the box. The top and bottom of the whiskers are the minimum and maximum, excluding outliers (defined 1.5 x IQR). The horizontal line inside the box represents the median value.

The groups differed in median RT during preTest (*p* < 0.001, *r* = 0.54; large effect size). CON was significantly faster (*Mdn* = 269.0 ms [IQR = 30.0]) in comparison to EXP (*Mdn* = 300.0 ms [IQR = 56.8]). The model fit improved significantly after adding group in the model, χ^*2*^ (1) = 11.10, *p* < 0.01. However, adding the interaction term did not further improve the model fit. Group was a significant predictor in the model [*F*(1, 41.32) = 12.03, *p* < 0.01]. EXP was 47.04 ms (± 13.56, 95% *CI* [20.46, 73.63]) slower than CON during both testing sessions (Figure 4).

#### Attentional lapses

The groups differed in the number of attentional lapses during preTest (*p* < 0.01, *r* = 0.47; moderate effect size). EXP had significantly more (*Mdn* = 8.5 [IQR = 14.8]) attentional lapses than the CON (*Mdn* = 1.0 [IQR = 2.0]). The model fit improved significantly after adding group in the model, χ^*2*^ (1) = 9.33, *p* < 0.01. However, adding the interaction term did not further improve the model fit. Group was a significant predictor in the model [*F*(1, 41.48) = 9.91, *p* < 0.01]. EXP had 8.10 (± 2.57, 95% *CI* [3.05–13.13) attentional lapses more than CON during both testing sessions (Figure 4).

### HR and HRV during the SART and PVT

#### HR during the SART

**Figure 5:**
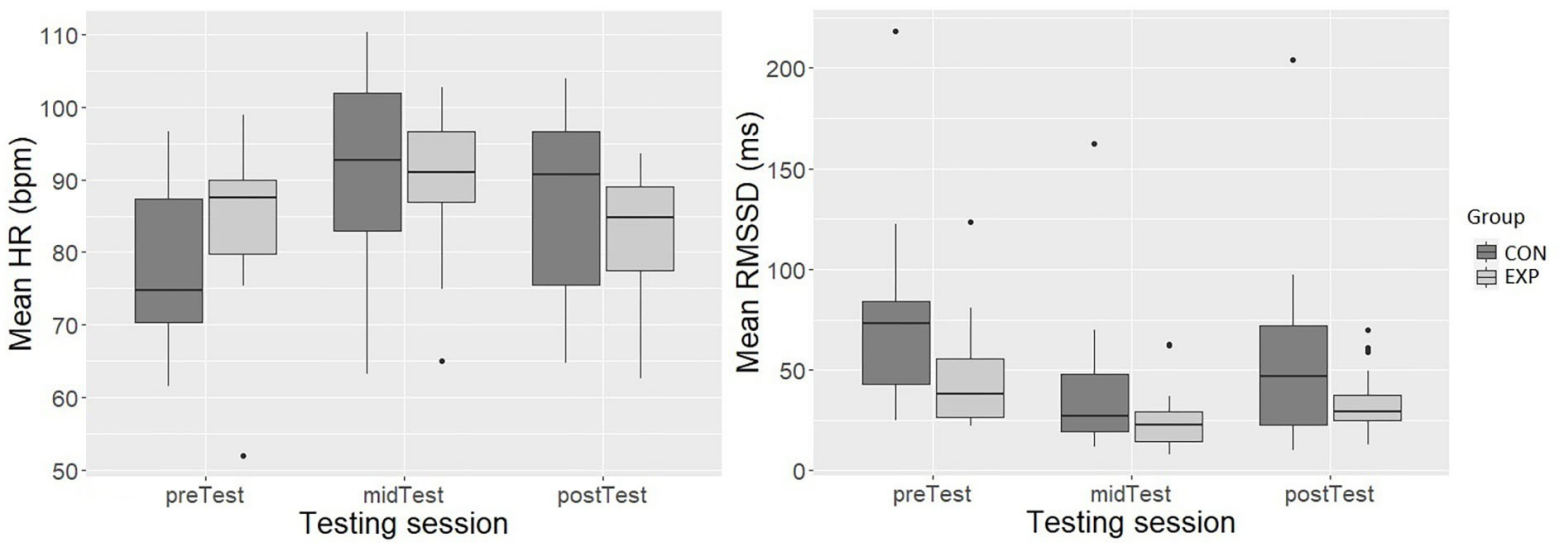
The mean HR (bpm) (y-axis; left-side graph) and the mean RMSSD (ms) (y-axis; right-side graph) during the SART testing sessions (x-axis; both graphs). The box depicts 50 % of all values (IQR). The top 25% and bottom 25% of all values are excluded from the box. The top and bottom of the whiskers are the minimum and maximum, excluding outliers (outliers defined 1.5 x IQR). The horizontal line inside the box represents the median value.

CON had a lower HR (*Mdn* = 74.7 bpm [IQR = 17.1]; *p* < 0.05, *r* = 0.34; moderate effect size) during preTest than EXP (*Mdn* = 87.5 bpm [IQR = 10.2]). Adding group into the model did not improve the model fit. The model with testing session as the only fixed effect was the final model. The testing session had a highly significant effect on the mean HR [*F*(2, 81.65) = 28.82, *p* < 0.001]. Mean HR was 9.26 bpm higher during the midTest (± 1.25, 95% *CI* [6.81, 11.71]) and 3.36 bpm during the postTest (± 1.25, 95% *CI* [0.91, 5.80]) compared with the preTest (Figure 5). *Post hoc* pairwise comparisons suggested that there was a 5.90 bpm (± 1.19, 95% *CI* [3.05, 8.76]) decrease in HR from the midTest to the postTest.

#### RMSSD during the SART

CON had a higher RMSSD (*Mdn* = 72.9 ms [IQR = 41.2]; *p* < 0.05, *r* = 0.40; moderate effect size) during the preTest than the EXP (*Mdn* = 38.0 ms [IQR = 28.6]). The model fit improved significantly after adding group in the model χ^*2*^ (1) = 5.39, *p* < 0.05. However, adding testing session and group interaction did not further improve the model fit. The testing session had a highly significant effect on RMSSD [*F*(2, 75.59) = 35.59, *p* < 0.001]. Similarly, the group had a significant effect on RMSSD [*F*(1, 43.55) = 5.47, *p* < 0.05]. The RMSSD decreased 27.31 ms (± 3.25, 95% *CI* [-33,68, −20.93]) during the midTest and 14.33 ms (± 3.28, 95% *CI* [-20.76, −7.90]) during the postTest compared to the preTest RMSSD. Furthermore, EXP had 19.98 ms (± 8.50, 95% *CI* [-36.71, −3.24]) lower RMSSD than CON over the three testing sessions (Figure 5). *Post hoc* pairwise comparisons indicated a 13.0 ms (± 2.94, 95% *CI* [-20.18, −5.77]) increase in RMSSD from the midTest to the postTest.

#### HR and RMSSD during the PVT

No statistically significant effects were found in either HR or HRV during the PVT.

### HR, HRV, and cognitive performance

#### HR and the SART

The categorized HR was marginally related to the EoC [*F*(1, 91.12) = 3.80, *p* < 0.1] when accounting for the variability of the group in the model. In the low HR group, there was marginally more EoC (−7.87% ± 4.04, 95% *CI* [-15.78, 0.05]) in comparison to the high HR group. The categorized HR variable was not related to the mean RT.

#### RMSSD and the SART

The categorized RMSSD variable did not predict the performance in SART EoC or mean RT.

#### HR and the PVT

**Figure 6:**
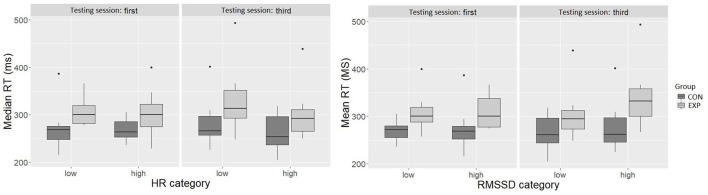
The relationship of HR (x-axis; left-side graph) and RMSSD (x-axis; right-side graph) categorical variables with median RTs (ms) (y-axis; both graphs). The box depicts 50% of all values (IQR). The top 25% and bottom 25% of all values are excluded from the box. The top and bottom of the whiskers are the minimum and maximum, excluding outliers (outliers defined 1.5 x IQR). The horizontal line inside the box represents the median value.

The categorized HR variable explained a significant amount of variability [*F*(1, 62.15) = 4.95, *p* < 0.05] in the model after accounting for the effect of group [*F*(1, 39.36) = 9.32, *p* < 0.01]. The group with low HR was 19.69 ms slower compared with the high HR group (± 8.85, 95% *CI* [-37.03,−2.35]) (Figure 6). However, the categorized HR variable was not related to the number of attentional lapses. *Post hoc* analysis showed that group and HR categorical variable interaction was not a significant predictor in the model. Hence, these results were not further investigated.

#### RMSSD and the PVT

Similarly, the categorized RMSSD variable explained a significant amount of variability in PVT median RT [*F*(1, 63.40) = 4.03, *p* < 0.05] when including group in the model, which was also a significant predictor in the model [*F*(1, 39.34) = 10.44, *p* < 0.01]. In the high RMSSD category, the median RT was 18.68 ms longer (± 9.30, 95% *CI* [0.45, 36.92]) than in the low RMSSD category (Figure 6). However, the categorized RMSSD variable was not related to the number of attentional lapses. The *post-hoc* analysis showed that the best-fitting model included RMSSD grouping variable and group interaction, and in addition, the testing session as fixed effects. In the model, RMSSD categorical variable [*F*(1, 52.32) = 7.66, *p* < 0.01], group [*F*(1, 37.25) = 9.39, *p* < 0.01], testing session [*F*(1, 31.82) = 6.87, *p* < 0.05], and RMSSD categorical variable and testing session interaction [*F*(1, 50.70) = 6.56, *p* < 0.05] were significant predictors. Pairwise comparisons using Tukey's HSD test showed that CON high RMSSD group was 64.70 ms (*SD* = 16.5) faster than EXP high RMSSD group (*t*(54.3) = −3.93, *p* < 0.01), and EXP low RMSSD was 45 ms (*SD* = 11.8) faster than the EXP high RMSSD during both testing sessions. In addition, the EXP low RMSSD group RT did not differ from the RT of CON low or high RMSSD groups.

## Discussion

The current study investigated the effect of the overnight military field exercise with sleep loss and high acute stress caused by military battle exercises on attentive abilities, response inhibition, and arousal. The main findings of the present study showed that the overnight training led to an impairment in upregulating arousal and attention. In contrast, sustained attention and response inhibition seemed to remain intact in a more stimulating and engaging task. Furthermore, the CQ battle led to a significant increase in stress in both groups, which in the end, negatively affected the attentional ability.

When tested before the CQ battle, the EXP participants' arousal and attentional state were impaired compared with the CON participants as expected (H1). Longer RTs and increased attentional lapses indicated slower psychomotor processing speed and increased attentional disengagement during the PVT. The finding is in line with the previous studies showing reduced activation in the frontoparietal sustained attention network and subcortical arousal and motor systems in conjunction with slow responses during continuous performance tasks under sleep loss (Hershey et al., 1991; Drummond et al., 2005)). Surprisingly, contrary to the expectation (H1), the overnight training did not affect sustained attention during the SART, as indicated by the equal RTs between the groups, and response inhibition, as indicated by the equal percentage of commission errors between the groups.

The acute stress caused by the CQ battle did not impair higher executive functions, such as response inhibition and sustained attention measured with the SART for either one of the groups. The ability of both the groups to inhibit responses remained constant throughout the three SART tests. However, in line with the hypothesis (H2), this happened at the expense of the response speed; the participants' psychomotor slowing became evident, but only after the CQ battle was over. This is consistent with research showing that fatigue reduces cortical activity after urban combat involving uncontrollability and unexpectedness (Suárez and Pérez, 2013), the same factors as in the current study. In addition, the CQ battle did not affect arousal, attentional ability, or attentional engagement with either one of the groups as measured with the PVT. Both groups performed at the same level before and after the CQ battle.

During the first SART test, as expected (H3), the SNS activation of the EXP group, as indicated by HR, and PNS withdrawal, as indicated by RMSSD, increased compared to the CON group which had a regular night of sleep. In addition, the CQ battle led to a significant increase in the SNS activation and PNS withdrawal during the second and third SART tests with both groups as hypothesized (H3). Furthermore, the SNS activation of all the participants decreased, and PNS activation increased from the second to the third test, indicating a partial recovery in the autonomic balance. As expected (H3), the EXP participants' PNS withdrawal was larger than the well-rested participants also during the second and third SART tests. However, no between-group differences in sympathetic activation were found during the second and third tests. Interestingly, RMSSD was the only index that differentiated the groups in each test. The recovery in the ANS balance was, on the one hand, likely due to the fact that the third SART test was conducted later after the CQ battle than the second, and on the other, possibly due to the higher intensity of the first track (Track A) compared with the second (Track B; Figure 2). The current findings, regarding between-group differences in the PNS activity, are supported by previous studies highlighting that RMSSD is a sensitive indicator of acute mental stress and workload (Botvinick et al., 2001; Mukherjee et al., 2011; Castaldo et al., 2015; Delliaux et al., 2019) and less sensitive to the other influences than the PNS activation (Hill et al., 2009).

Surprisingly, contrary to the hypothesis (H3), no overnight training or CQ battle effect on the ANS activity was detected during the PVT. During the last PVT, after completing both CQ battle tracks, the ANS activity of both groups regressed to the pre-CQ battle level. It should be highlighted that the PVT was the final test administered 30 min after the CQ battle was over. This was the most likely reason for the shift from the sympathetic dominance, which was evident during the SART, toward an increased vagally mediated HRV, i.e., lower arousal. In contrast to the findings regarding the ANS activity during the SART, the groups did not differ in the ANS activity during the PVT tests, suggesting test-related effects.

Finally, we divided the participants into two groups based on the median of the PNS activity (low and high RMSSD) and the median of the SNS activity (low and high HR) to test the last hypothesis (H4). As expected, the participants with below-median PNS activation (low RMSSD) had higher arousal and alertness than those with the above-median PNS activation (high RMSSD) as measured with the PVT (i.e., had the longest RTs). Similarly, the above-median SNS excitation (high HR) participants had higher arousal and alertness than those with below-median excitation (low HR). Hence, the more relaxed participants, evident in HR and HRV, were less aroused and alert, as indicated by the PVT RT. Interestingly, the *post-hoc* tests indicated that the more alert EXP participants (low RMSSD) had comparable arousal and alertness with the low and high PNS activity CON participants. Only the EXP participants with high PNS activity (high RMSSD) had significantly decreased arousal and alertness compared with the low PNS activity EXP and CON and high PNS activity CON participants. However, the ANS activity was not related to attentional disengagement (i.e., attention lapses). In other words, despite comparable arousal between the low PNS activation EXP participants and both low and high PNS activity CON participants, the low PNS activity EXP participants continued to have significantly more fluctuation in their alertness compared with CON participants. Contrary to expected (H4), the ANS activity was unrelated to the SART performance.

In the upcoming sections, we will discuss the results found in the study more in-depth. First, we go through the results regarding the differences between the SART and PVT performance in conjunction with the HR parameters. Second, we focus on the expectation that the battle-induced arousal protects the post-overnight training performance.

The findings regarding the SART and PVT performance in conjunction with the ANS activity during the tests may relate to differences in groups' attentional effort allocation. According to the attentional control theory, one can overcome a lowered ability to perform by exerting an additional, compensatory effort (Eysenck et al., 2007). The investment of effort is associated with a decrease in HRV and an increase in HR (Mukherjee et al., 2011; Fairclough and Mulder, 2012). Thus, based on this view, the EXP conscripts exerted additional mental effort during the SART but not during the PVT. Motivation to perform may have been the underlying factor, which is also related to an increase in HR and a decrease in HRV (Fairclough and Mulder, 2012). Interestingly, Massar et al. (2019) found that rewards improved vigilance in the PVT in participants with sleep loss. Therefore, the performance impairment in the PVT is not solely due to fatigue but also due to lack of motivation. It should be noted that motivation, effort allocation, and stress are not mutually exclusive or even entirely distinct concepts (especially in the framework of cardiac activity): motivation, stress, and effort are closely related and often coupled with increased SNS activity in conjunction with the PNS withdrawal (Mukherjee et al., 2011; Fairclough and Mulder, 2012; Massar et al., 2019). One crucial difference between the SART and PVT is that in the SART, one can monitor the success level. When one makes the inhibition error, the program prompts an error message, which can motivate to increase effort. In addition, the PVT is longer in duration (~10 min) than the SART (~5 min), and the performer does not have any reference against which to evaluate the performance. Lengthy, monotonous, and even subjectively boring PVT does not motivate an effortful performance, particularly considering that one cannot judge the performance as sufficient or correct. The findings indicate that the overnight training can reduce efficiency, not necessarily effectiveness when performing tasks requiring sustained attention and response inhibition. In contrast, in a long and monotonous, less engaging vigilance task, the overnight training results in a performance impairment.

The findings above suggest that the intact performance of the participants with sleep loss is dependent on a bottom-up (i.e., stimulus-driven) activation. In a normal wake state, the locus coeruleus noradrenergic projections to the cerebral cortex maintain a sufficient arousal level (Chandler et al., 2014; Hudson et al., 2020b). In addition, this norepinephrine system is closely related to the activation of the frontoparietal attention network (Posner and Petersen, 1990; O'Halloran et al., 2014). However, sleep loss-related fatigue impairs the arousal mechanism and reduces the energy to upregulate attention and effort (Engle-Friedman, 2014; Van Dort, 2016). Monotonous low stimulus intensity tasks propagate this effect leading to a further decrement in alertness and increased fluctuation in attention (Drummond et al., 2005). In contrast, arousing task properties can protect the low stimulated state supporting alerting attention toward the task (Sara and Bouret, 2012). This is particularly the case with motivating stimulus factors, such as rewards and punishments, and when the stimuli occur infrequently and unexpectedly (Bouret and Sara, 2005). Hence, due to the exogenously arousing nature of the more active SART, task engagement and effort allocation of the participants with sleep loss are supported compared to monotonous and less engaging PVT, in which the performance becomes impaired.

The observed psychomotor slowing in the SART after the CQ battle suggests that impairment in sustained attention may become evident after prolonged acute stress and effort allocation. The psychomotor slowing combined with intact response inhibition reflects the observed speed-accuracy trade-off effect. In other words, the slowing allowed the participants to refrain from responding to the inhibition trials. A few findings may shed some light on this phenomenon. First, there was no slowing in the PVT, which according to McIntire et al. (2014), is simply a test of arousal, not one of vigilance. Thus, the SART slowing was apparently not due to a decreased arousal or general motor slowing *per se*. Second, neither slowing was a primary effect of high acute stress (e.g., a strategy to overcome stress-induced habituated responding, i.e., the problem with response inhibition) as the slowing was not present after the first track. In addition, the co-occurrence of a high error rate with slow motor responses reflects slow processing speed or lapsing attention (O'Halloran et al., 2014). Thus, the findings may reflect the ones by Suárez and Pérez (2013). According to them, high acute stress in combat exercise leads to fatigue, impairing information processing. It is possible that, after a prolonged effortful cognitive processing and stress regulation, the psychomotor slowing may relate to a slowing in information processing speed, perhaps due to a fatigue-related impairment in attention.

The expectation (H4) that the stimulating nature of the CQ battle would protect the EXP participants' arousal and attentive state was not fully supported. This was probably due to the fact that ANS activity returned to pre-battle levels during the PVT after the CQ battle. However, we obtained some support for the expectation (H4) that the sleep-deprived individuals would benefit from the CQ battle-induced arousal increase during a simple vigilance task. The findings indicated that the EXP participants with low PNS activity have increased alertness and attentive ability compared to the EXP participants with high PNS activity. However, the effect was observed before and after the CQ battle. Therefore, it is likely that the excitement or tension related to the battle exercise may have led to a parasympathetic withdrawal among some EXP participants. Still, we found a significant decrement in attentional engagement even among these more alert participants. In other words, despite the ability for producing equally fast psychomotor responses, they continued having a large number of attentional lapses. However, the current study cannot disentangle why some EXP participants had low PNS activation and increased wakefulness. The findings from Chua et al. (2014) may provide one possible explanation. According to their study, individuals with low baseline HRV and lesser variation in PVT RT tend to be less vulnerable to the effects of sleep loss (Chua et al., 2014). However, more research is needed to better understand the individual differences that may protect against sleep loss-related adverse effects on arousal and attentive ability.

There are some limitations to the study. First, it is possible that the sample size of 45 was not sufficient to reveal the between-group effects in SART. This is, however, unlikely, as other studies have shown significant within- and between-group effects for SART with even smaller sample sizes (Rabat et al., 2019; Magnuson et al., 2022). Power calculations are recommended for future studies to determine the appropriate sample size *a priori*. Second, the first team of EXP started the CQ battle at 7:20 a.m., while the first team of CON started their CQ battle 1 h later, at 8:25 a.m. In line with the starting time, the last EXP team finished the CQ battle training at 3:48 p.m., and the last CON team finished the CQ battle at 5:03 p.m. Consequently, the groups did not perfectly match with respect to their circadian phase; there was a difference of approximately 1 h in the morning and 1 h in the afternoon between the study groups. Altogether five CON teams and five EXP teams were perfectly matched concerning time. As the alertness level is known to be influenced by both the time spent awake and the circadian phase (Åkerstedt and Folkard, 1997; Lo et al., 2012), this difference in timing may have affected some of the results. According to Lo et al. (2012), the circadian effect concerns mainly the PVT but not SART. Yet, the effect of a 1-h difference should only have a very small effect, particularly in comparison to the effect of time awake (Åkerstedt and Folkard, 1997). However, as we cannot fully exclude the possibility of some circadian rhythm-related effects on cognitive performance and physiological measurements, this needs to be considered when evaluating the findings. Finally, sometimes, when cognitive tests are repeated in close succession, the participants may improve their performance either due to familiarity with the test or as a consequence of developing better strategies to perform it. In this study, the participants were instructed to balance speed and accuracy equally to avoid variation in the response strategy. Also, they were familiarized with SART on day 1 before the CQ battle took place, on days 2 and 3 to avoid the practice effect, which could be strongest in the first repetition of the test. However, according to the findings from Robertson et al. (1997), SART test-retest reliability is high. For PVT, this is not a concern, as it is known to be free from practice effects (Dorrian et al., 2004; Lim and Dinges, 2008), and the strategy is also straightforward and not subject to variation.

### Practical implications

The difficulty to upregulate arousal in the face of monotonous tasks lacking top-down stimulation appears to be an essential factor behind the EXP participants' cognitive impairment. Drummond et al. (2005) have shown that individuals suffering from sleep loss show a shift from the activation of the sustained attention network toward the cortical midline structures. The activation in these areas is associated with the default mode network activity (Drummond et al., 2005) suggesting that psychomotor slowing is related to mind-wandering and task disengagement. This shift in attention from the ongoing task can have implications for natural work conditions. It becomes increasingly difficult to notice meaningful changes in the environment timely, which at worst, leads to severe consequences for health and wellbeing. Radar monitoring, for example, is such a task in which the performance of individuals with sleep loss may decrease considerably. In contrast, more active sustained attention and response inhibition tasks that provide sufficient exogenous stimulation reinforce optimal performance for a longer period of time. As a result, the EXP participants perform as well as their well-rested counterparts. Hence, the participants who lack internal resources to upregulate arousal due to fatigue performance ability can be adequate in, for example, shoot-decision tasks provided that the conditions are stimulating and arousing. Therefore, studying the effect of external stimulation, such as caffeine and adenosine receptor antagonists (Samuels and Szabadi, 2008) for comprehensive review) and vagal nerve stimulation (McIntire et al., 2021) to protect the cognitive performance of the participants with sleep loss during monotonous tasks in ecologically valid settings could be incorporated into the future research. In case these are found to counteract the harmful effects of sleep loss, the usage of these methods would be worthwhile to consider when the placement of the individuals lacking energetic resources in monotonous low-intensity tasks cannot be avoided.

A significant between-group difference was found more often in RMSSD than in HR. The results showed that while HR of both groups may have been equally high, the EXP participants had a significantly decreased RMSSD compared to the CON participants. Hence, it is likely that HR is more confounded with factors, such as physical movement. In other words, HR does not exclusively reflect, for example, the activity of the SNS. Therefore, future applications and studies might benefit from using RMSSD rather than HR as an index of an alternated activity of the two branches of the ANS.

## Conclusions

In conclusion, the present study observed that individuals deprived of sleep could not maintain sufficient arousal levels for consistent cognitive performance, especially in monotonous tasks lacking external stimulation. However, this was not the case with a shorter, more active, and engaging vigilance task with immediate feedback. Hence, monotonous tasks that require a certain level of alertness to notice possible meaningful changes may not be optimal for fatigued individuals. In these types of tasks, such as radar monitoring, individuals lacking energetic resources may perform poorly. However, no impairment due to the overnight training or high acute stress is seen when performing more active tasks, such as making decisions about whether or not to shoot. This presumably relates to high motivational control in tasks requiring response inhibition and vigilance and in which the performance can be evaluated due to direct feedback. However, after prolonged acute stress, the ability to maintain sustained attention may show deterioration manifesting as a slowing down of reactions (i.e., a longer response time). Furthermore, it is reasonable to assume that some individuals may be more vulnerable to the effects of sleep loss. In addition, it is concluded that such CQ battle can be used to induce high acute stress. Finally, this study found that PNS withdrawal is dependent on and possibly related to effort allocation/motivation and task type. RMSSD is more sensitive than HR in indicating changes in effort/motivation/stress.

## Data availability statement

The participants have not given their consent to publish their data. However, the data can be made available for inspection if requested. Requests to access these datasets should be directed to tomi.passi@ttl.fi, satu.pakarinen@ttl.fi.

## Ethics statement

The studies involving human participants were reviewed and approved by Ethics Committee of the Hospital District of Helsinki and Uusimaa and Finnish Defense Forces. The patients/participants provided their written informed consent to participate in this study.

## Author contributions

TP: study conception, methodology, formal analysis, investigation, data collection, data curation, and manuscript preparation—writing the initial draft. SP: study conception, methodology, investigation, data collection, writing—review and editing, funding acquisition, supervision, and project administration. KL: data curation, investigation, study conception, writing—review and editing, and data collection. JL: investigation, data collection, supervision, project administration, funding acquisition, writing—review and editing. JN: investigation, data collection, project administration, and writing—review and editing. JR: investigation, data collection, and data curation. JV, KP, KK, and TO: resources and writing—review and editing. SM: writing—review and editing. All authors contributed to the article and approved the submitted version.

## Funding

This study was funded by the Finnish Defence Forces (AQ9520) as a part of a four-year research and development program.

## Conflict of interest

Authors JL and JN are employed by VTT Technical Research Centre of Finland Ltd. Author JR is employed by Savox Communications Oy Ab. The remaining authors declare that the research was conducted in the absence of any commercial or financial relationship that could be construed as a potential conflict of interest.

## Publisher's note

All claims expressed in this article are solely those of the authors and do not necessarily represent those of their affiliated organizations, or those of the publisher, the editors and the reviewers. Any product that may be evaluated in this article, or claim that may be made by its manufacturer, is not guaranteed or endorsed by the publisher.
